# A Few Practical Remarks on Counter-Irritants, and Their Effects as Remedial Agents

**Published:** 1830-03

**Authors:** Anthony Todd Thomson


### COUNTER-IRRITANTS.

*A few Practical Remarks on Counter-Irritants, and their Effects as Remedial Agents.* By Anthony Todd Thomson, M.D. &c.

Counter-irritants comprehend various species of remedial agents, many of which have been known and employed from a very early period. They are intended to relieve deep-seated local affections, and also local symptoms of general affections, by exciting certain impressions on the surface of the body. Whether they operate by producing a conversion of disease, or rather by exciting a new diseased action, so that that which already existed is suspended or destroyed, on the principle that two diseases cannot be present in the body at the same time? or whether the benefit derived from them depends on a change in the local momentum of the blood? are questions which it is not my intention, at present, to examine. My object, in this paper, is to lay before the profession some results of my experience of the advantages to be expected from the application of counter-irritants, in diseases which have, in almost every instance as far as my information extends, proved fatal. Before proceeding, however, with the detail of these cases, I may, perhaps, be permitted to offer a few remarks upon the effects of counter-irritants on the parts to which they are applied, and the comparative advantages to be expected from the different kinds of counter-irritants that have been employed.

Under the term counter-irritants are included four varieties of external applications: *rubefacients*, *vesicants*, *pustulants* (if I may be allowed to employ such an expression), and *caustics*.

The substances comprehended under the term *rubefacients*, I need hardly remark, produce an increased action, or temporary inflammation, in the superficial vessels of the part to which they are applied, attended by a sensation of heat, and generally of pain. They have been found useful in relieving local pains, when no organic derangement is supposed to exist; but their application, in some painful affections, such as acute rheumatism and gout, when much general febrile excitement is present, has occasionally been followed by fatal metastasis.

*Vesicants* have been found more extensively useful than rubefacients, in the same range of diseases; and, whatever may be the cause, their application in acute affections has been less frequently followed by metastasis, than when rubefacients have been used. From the large quantity of serum which is thrown out between the cutis vera and the cuticle during the operation of a vesicant, there is little doubt that the increased action which they excite extends beyond the superficial vessels of the part to which they are applied; and, thence, their curative effects are more certain than those of rubefacients; but why they are less frequently followed by metastasis, is not so readily explained.*

* If I might hazard an opinion, I should say that it is owing to the more complete, and consequently more permanent, impression made by blisters; whereas the impression made by rubefacients is transitory, and although it, as it were, unsettles the internal disease, yet, its influence rapidly passing away,

Under *pustulants*, I include those substances, the application of which to the skin is followed by suppurating phlegmons, or pustules. The best known of these substances is the Tartar-emetic ointment. I have employed it for several years in many cases, and have never seen it followed by metastasis, or by other untoward circumstances. But it is slow in producing its effects; and, thence, is less calculated for acute cases of disease than vesicants. Without stopping to examine the correctness of the opinion of some continental physicians, that it acts in relieving pulmonary inflammations, by some specific and direct action on the lungs, independent of its operation as a counter-irritant, I may state that my observations induce me to recommend it as peculiarly adapted for phthisical cases, when there is reason to believe that ulceration has not yet taken place.*

The fourth kind of counter-irritants, *caustics*, operate by destroying the life of the skin to which they are applied, producing a slough which separates and is thrown off from the surrounding living parts, and which leaves behind it a suppurating sore, that may be kept discharging for a considerable time, by the application of irritating substances to the raw surface.

These are the most powerful description of counter-irritants,† the most permanent in their effects, and I may venture to assert, those on which the practitioner may place some reliance for the relief of deep-seated pains, even when connected with organic affections.

There are two species of caustics, the *actual cautery*, which destroys the part to which it is applied by the sudden excitement of an action in it greater than its vitality can sustain; and the *potential cautery*, which comprehends chemical substances that destroy the part to which they are applied by breaking those affinities between the components of the living solid which are maintained by its vitality, and producing new, which are incompatible with its living state. I have selected the three following cases which I am about to describe, as illustrative of both species of caustic counter-irritants.

Case I. J. S——, ætatis forty, a watchman, was adthe diseased action is left to fall upon any other part of the system. I am aware of the mechanical nature of this explanation; but I cannot conceive any other that so readily explains these effects of vesicants.

* When a quicker effect is required, I have found the following ointment adequate to the production of pustules: ℞. Acidi Sulph. F. ʒss.; Acidi Acetici fort. ʒiss.; Ung. Cetacei ℥iss. tere optime; mag. glandis thoraci nocte maneque applic.

mitted a patient at the Chelsea and Brompton Dispensary. His disease was a large fungous tumor, which protruded from under the lower edge of the greater pectoral muscle of the right side. It had penetrated the skin by ulceration; and, at the time of his admission at the Dispensary, it presented the aspect of a lobulated tumor, of a dark red or purple colour, about the size of the fist of a full-grown man, compressible, and bleeding on being slightly handled. It appeared to extend upwards beneath the muscle, and to take its origin, by a narrow neck, from the clavicle, or from some part near that bone. There was also another tumor, about the size of a large walnut, seated in the side of the thorax, two inches below the axilla: this had not penetrated the skin, but caused considerable pain when it was touched or pressed by the arm. The man was in an emaciated condition; his countenance expressive of great anxiety and suffering, and his complexion displaying that sallow hue which is almost pathognomonic of cancer. The tongue was furred; the pulse quick and irritable; the skin dry and harsh; and the urine scanty, high coloured, and depositing a pinky sediment; whilst the bowels were in the most irregular state. The patient enjoyed little sleep, owing to a diurnal fever that made its attack in the evening, and terminated in profuse sweating in the morning. He had been unable to perform his duties as a watchman, for some weeks before he applied at the Dispensary.

In tracing the history of this disease, he informed me that the tumor commenced about two years before, when he was a soldier. That it first attracted his attention when it was about the size of a hazelnut; that, as it slowly increased, extending downwards, it raised the pectoral muscle; but he did not apply to the regimental surgeon as long as he was able to perform his duty, which was the case until the skin began to ulcerate and the tumor to protrude from beneath the edge of the muscle. He was then sent to the hospital at Chatham, where the tumor was partially extirpated; but he suffered greatly from the hemorrhage that followed the operation; and, as soon as the wound healed, he was discharged from the service, and admitted an out-pensioner of Chelsea hospital. Before this time, however, the tumor had again considerably enlarged, and it had again penetrated the skin at the time he commenced the office of a watchman.

The examination of the tumor convinced me, that it was of that species which has been termed Fungous Hæmatodes; being, as I have already stated, irregularly lobulated, or of a cauliflower form, of a deep red colour, spongy, bleeding freely when slightly touched, and excreting a thin acrid fluid, which excoriated the surrounding sound skin.

To use the knife again was out of the question; I resolved, therefore, to destroy the tumor by the actual cautery; but, before determining finally on that method of proceeding, I sent the patient to my friend Mr. Guthrie, who approved of my proposal to destroy it by the cautery; and the man having agreed also to the operation, it was performed the next morning, in the following manner. Four folds of thick brown packing paper, cut into a circular shape, of a size sufficient to cover the whole of the pectoral muscle and part of the side of the body, with a hole in the centre, exactly the size and form of the tumor, were soaked in water, and afterwards pressed so as to squeeze out all the superfluous fluid. This cool cover was fixed over the seat of the disease by two turns of a roller, which went round the body, the tumor only being exposed; and an iron spatula, such as is usually employed for spreading plasters, heated to whiteness, was applied with considerable pressure over the whole surface of the tumor. The hot iron was held on the part longer than a minute; but the pain was so supportable, that the patient, on being questioned how long he could have borne it, replied five minutes.

He walked home after the operation, which, as might be expected, was not followed by any bleeding; and in the evening he poulticed the cauterized part, as he had been directed. In less than twenty-four hours, the slough began to separate, and, in three days, the whole of the tumor seemed, as it were, to dissolve away from its origin, which led me to hope that it was completely destroyed. Much constitutional derangement accompanied this process, and the free administration of bark, sulphuric acid, opium, and wine, was requisite to prevent the patient from sinking under it. In three weeks his general health was restored, and the wound was healed in another week. So completely, indeed, had every vestige of the tumor disappeared, that I exhibited the patient at a meeting of the Medico-Chirurgical Society, as an instance of Fungous Hæmatodes cured by the use of the actual cautery.

The smaller tumor, in the axilla, was now extirpated by the knife; and the man returned to the performance of his duties as a watchman.

Six months afterwards, this patient again presented himself at the dispensary, the tumor having once more showed itself under the pectoral muscle. At this time it was about the size of a small apple, and pushed forward the muscle under which it was seated, but without any approach to ulceration in the integuments. I now determined to try the power of the cautery as a counter-irritant; and, with that intention, applied a smaller iron than that which was used on the former occasion over the tumefied part of the muscle, with a degree of pressure not more than sufficient to produce a slough penetrating very little deeper than the cuticle. The result exceeded my expectations: the tumor in a few days appeared considerably diminished; and by repeating the cauterization twice in the space of twelve days, it almost disappeared.*

In this manner, by the occasional use of the cautery, the tumor was kept of a very small size for the space of twenty months, during which period the man enjoyed excellent health, and was capable of performing his duties as a watchman. Some domestic troubles, the illness of his wife, and a habit of drinking into which he had fallen, prevented him from coming to me for some months; and, when I again saw him, the tumor was beginning to reappear under the edge of the muscle, and ulceration of the skin had already commenced.

As I could not apply the cautery on that day, he was directed to return on the following; but I saw him no more until he was on his deathbed, eleven months afterwards, when his wife called and requested me to see him. The poor man died in less than a week afterwards. I was informed by his wife that he had put himself under the care of some quack, who promised him a speedy cure without the use of the cautery; and that a sense of shame, on finding he had been duped, prevented him from again applying to me, until the disease had undermined the powers of his constitution, and he was sensible of his approaching dissolution.

On dissecting out the tumor, after the death of the patient, it was found to originate, as I had anticipated, from the under part of the clavicle, and to stretch down between the pectoralis major and minor muscles. No corresponding internal disease was found in this case, although it has been frequently observed in cases of Fungus Hæmatodes, except a small warty tumor on the pleura costalis, immediately under the seat of the tumor. This, had the life of the patient been longer preserved, might have increased and propagated the disease in the cavity of the thorax.

In remarking on this case, it may be justly said that there was no novelty in the application of the actual cautery to the fungus; for, although this remedy had long been dis-

* In all these applications of the hot iron, the parts were protected from radiation by the moistened paper.

used in England, yet it is of great antiquity, having been used by Hippocrates, Celsus, Albucasis, and other ancients, and more recently, in similar affections, by Pouteau, Percy, Larrey, and Maunoir, on the continent. I am not aware, however, of any instance on record in which the cautery was employed as a counter-irritant, in the manner in which I have described; and I trust I am not mistaken in supposing that the knowledge of this fact may lead to very important improvements in practice, although I have no satisfactory rationale of the operation to offer. That the same beneficial effects would result from the application of the mineral caustic, is not impossible; but I am inclined to think that its power is much less efficacious: and I infer this from the instantaneous and very powerful effect produced on the application of a whitehot iron to the skin, and the rapid separation of the slough. Pure Potassa does not act so immediately, and the slough requires at least four times the period to separate. The mineral acids and other caustics are equally slow in their effects. Moxa has been much used on the continent, instead of the hot iron, from the idea that it is a less barbarous application; but I am inclined to think that any individual who would submit to a trial of the two remedies, provided that the iron be applied when it is at a white heat, and the effects of the radiated heat be guarded against by the method I have described, would not hesitate to prefer the hot iron to the moxa. The death of the part is instantaneous, and the influence of the caloric not being felt beyond the spot which the iron touches, no more inflammation is excited than is requisite for the separation of the slough. I have had frequent opportunities of employing the actual cautery as a counter-irritant, since the occurrence of this case; and, even when women were the subjects of the operation, the application of the iron has been submitted to a second, a third, and a fourth time, without any hesitation.

Case II. About two years ago, I was requested to see the Rev. Mr. W—, a clergyman of the church of England, ætatis sixty-eight. He had been ill for upwards of a year previous to my advice being requested, and was confined to his sofa, under the impression that the spine was affected. In tracing the history of the attack, I found that, several years before the commencement of the disease under which he was labouring, he had been thrown from his horse; that the symptoms of his disease soon afterwards appeared, and had slowly but progressively advanced. He complained of numbness in the upper extremities, with the sensation of great tightness across the chest; the lower extremities also were less under control than usual; and the bowels were so torpid, that evacuations were procured only by artificial means. Daily, after dinner, the hands became hot, and at length burning; the breathing a little oppressed; and the susceptibility of touch so sensitive, that moderate friction with gently stimulant embrocations could not be endured. His intellect was entire, although occasionally some degree of fulness and throbbing were felt in the temples. He slept well, and his appetite, although irregular, was moderate. No means that could be devised had yet succeeded in promoting perspiration.

On examining the state of the vertebral column with close attention, I could not perceive the smallest indication of disease in that chain of bones, and consequently extended my inquiries as to the state of the medulla and its membranes. The result of these was a conviction that there was some affection of the cord: either that state which the French have termed ramollissement, or a thickening of the theca. In the event of either state being the cause of the symptoms under which Mr. W. was labouring, it was evident that no necessity existed for confining him to the horizontal position: he was therefore desired to sit up as much as his strength would permit him; to take gentle exercise, with the aid of a crutch or the arm of another person; for a long confinement to the horizontal position had so debilitated his frame, that he could scarcely sustain the upright position for half an hour at a time. His bowels were attended to, and the secretions corrected by a mild alterative course of medicine; and this, in conjunction with gentle tonics, was continued until the tone of the frame was restored, the appetite improved, and he could sit up all day.

It is unnecessary to detail the means that were devised at different times to remove this disease; but little permanent benefit was affected until the applicaticn of the actual cautery on each side of the vertebral column, in the loins. It was effected by means of a double-headed iron, the heads or buttons being about the size of half-a-crown each, and about three inches asunder. The part being guarded as described in the former case, the iron was heated to whiteness, and applied by my colleague Mr. Pattison, in such a manner that both buttons acted at the same time. Little pain was experienced, and that was lessened by the application of diluted Liquor Ammoniæ to the surrounding skin. In forty-eight hours the sloughs separated, and a moderate discharge was maintained for about six weeks, after which the ulcers were allowed to heal.

The change that followed the use of the cautery in this case was soon very apparent. No medicine was given, except to regulate the bowels; and although he is still weak, and complains of some degree of numbness of the upper extremities, yet he is comparatively well, and is capable of taking walking exercise, in a degree sufficient to preserve his health. Moxas had previously been employed, but without any decided advantage. He has lately removed to Hastings for the benefit of a mild climate during the winter months.

Case III. R. M—, esq a merchant in Liverpool, ætatis thirty-four, had occasionally consulted me by letter, previous to the 3d of January, 1812, when he came to London, and placed himself under my care. Being a relation of my wife, I received him into my house; and, consequently, had many opportunities of closely watching both the symptoms of his disease and the effects of the remedies employed to relieve them.

Mr. M.'s state of health previous to his arrival in London had afforded a strong suspicion that the kidneys, or rather one of them, contained calculous matter, sufficient to excite a great degree of irritation in the viscus. He suffered from continual pain in the loins, accompanied with profuse perspiration, and the urine deposited a large quantity of ropy mucus. Mr. Cline, whom he had, also, consulted, when he was in London, in the spring of 1810, was of the same opinion as myself respecting the state of the kidney; and the plan he recommended was to soothe the general habit, and thus, by allaying irritation, to allow nature the opportunity to restore the healthy secretion of the urine. He advised, with this view, a temperate diet, bland demulcent drink, the avoiding wine and all other causes of excitement; and, for medicine, large doses of powder of Sarsaparilla, with carbonate of soda, and at the same time the tartrate of iron, with extract of Sarsaparilla, to be taken in moderate doses. This plan was pursued until half an ounce of Subcarbonate of Soda, and four drachms and a half of the powder of Sarsaparilla, were daily swallowed, but with no evident advantage: on the contrary, the unfavorable symptoms increased; and, when Mr. M. arrived in London, a swelling had appeared in the region of the kidney, protruding forwards, and occasioning an evident external tumor in the right hypochondrium.

It is unnecessary to detail the various plans which were adopted for the relief of Mr. M. During the four months which he remained with me, he had seen Dr. Baillie, who, as well as Mr. Cline, objected to the introduction of caustics on the loins, which I had more than once suggested. He returned, therefore, to Liverpool, with the tumor in the region of the kidney rather larger than when he arrived in town. In this state he continued for several months, until, having been thrown from his horse, and fallen on the affected side upon a large stone, the tumor became so painful, and increased so rapidly in size, that he returned to London, and consented to the use of the caustics. As my plan, however, had been objected to both by Mr. Cline and Dr. Baillie, I urged Mr. M., before it was commenced, to see Dr. Henry Ainslie, who was requested to meet me in consultation on the 28th of September. On examining the tumor, which was now apparently as large as a man's head, Dr. Ainslie agreed with me that it was an enlarged kidney, and that the employment of counter-irritants, in the manner I had suggested, afforded the best prospect of relief; while, at the same time, he recommended the administration of iron and bitters, with the view of keeping up the general strength, which was much reduced, and restoring the tone of the diseased organ.

Two large caustics, composed of the Pure Potassa, were now applied upon the loins, about five inches in length and one in breadth; and, as soon as these were in a state to receive a pledget of lint smeared with Savine ointment, two others were opened; and in this manner the whole right side, from the spine to the umbilicus, was successively treated; four issues being always open at one time, two discharging freely, and two in a state of preparation. As soon as the first issues were formed in the loins, the most evident benefit was experienced: the pain and uneasiness in the region of the kidney diminished; the nocturnal perspirations ceased, and the febrile sensation of fatigue on rising in the morning was soon removed. The deposition of mucus in the urine, however, still continued to be very copious; and although the odour of that secretion, and its colour, which had been previously very unnatural, were improving, yet little ground was gained in this respect for many weeks. The tonics had been discontinued; and, on Dr. Baillie being again consulted, he now approved of the caustics; and recommended the perineum also to be blistered, under the idea that the prostate gland was in a state of disease, and the chief source of the ropy, mucous deposition in the urine. He also recommended a scruple of alum and four grains of extract of opium to be taken daily.

Much benefit was derived from this change in the plan of treatment; and when Mr. M. left London for Liverpool, towards the end of the summer, the tumor was reduced to nearly one half the size it was before the caustics were applied; his general health was greatly improved, and the urine was nearly free of mucus.

In a few months the issues were healed; and I saw nothing of Mr. M. until the spring of 1817, when he again came to London, the tumor having begun once more to enlarge. When I saw him, it was not so large as when the caustics were first introduced, but it was much larger than when he left me in 1814. The same plan was pursued, with the same beneficial results; and, by a letter dated the 21st of June, 1817, I find that he left me much improved, and that I had given him the following instruction respecting his future management of himself.

"With regard to medicines, I would advise the continuance of those which you have been lately taking, until you find the stomach and bowels act in a more regular and natural manner. If costiveness intervene, the medicines must be omitted for two days, and the bowels be opened with a scruple of Rhubarb. The issues must be kept up for more than a year. Indeed, it is impossible to name any period at which you will be able to do without them; but you must regard them as the sheet-anchor of your hope of recovery, and avoid leaving them off before the kidney be fully reduced. Those which are now open should be closed soon after you get to Liverpool, and two others opened near them, by means of the Causticum acerimum; and in this manner they should every now and then be changed, for I place more confidence in the repetition of the counter-irritation which they produce, than in any discharge from them. If you could bring your mind to submit to the actual cautery, it should be tried. I have great expectations of benefit from so powerful an agent. To remove every source of sympathetic irritation in the urinary organs, let a full-sized bougie be passed into the urethra once or twice a month. I cannot commit myself by promising you perfect health by the steady pursuance of this plan; but from the result of the plan as it has already been tried, there is the greatest probability of a near approximation to health."

Mr. M. did not live to realize these hopes, owing to an attack of pulmonary inflammation, which proved fatal on the 27th of November, in this year. As his death occurred at Liverpool, I shall give the account of it, and the post-mortem examination, in the words of Dr. TRAILL, who, assisted by Dr. VOSE and Dr. RUTTER, attended him in his last illness.

"The steady perseverance," says Dr. Traill, "in the use of large caustic issues, three of which were constantly kept in an active state, produced a sensible diminution of the size of the tumor; and Mr. M.'s great attention to diet, and regularity of exercise, effected such an amelioration of all his complaints, that his state became comfortable, and promised a progressive improvement.

"Early in November last, after a week of slight indisposition, Mr. M. imprudently exposed himself in a long ride in an open gig. He returned home chilled, and, after ineffectual efforts to regain his natural warmth, retired to bed. When I first saw him, about ten o'clock on the same evening, his respiration was quick and laborious; he complained of a very painful constriction of the chest; his skin was warm and moist, and his pulse soft, small, and 140 in a minute. A large sinapism was immediately applied to the chest, and his feet and legs were wrapped in flannels wet with hot vinegar, in which mustard had been mixed. He took a draught, with Camphor mixture, solution of Acetate of Ammonia, and sweet Spirit of Nitre. At one o'clock the same night, his breathing was a little easier, and he perspired freely.

On November the 17th, at seven A.M., Mr. M. passed the remainder of the night nearly as I left him, and was not sensibly better in the evening. The Pil. Scillæ cum Calomelane et Ipecacuanhæ was ordered.

"As it now became an important question whether relief might be afforded by bleeding, I requested a consultation with Dr. Rutter. At nine A.M. from six to eight ounces were ordered to be taken from the chest by cupping. After six ounces were taken, no relief being obtained, and the pulse becoming more feeble, the bleeding was carried no further. A large blister was applied to the chest. In the evening, we found Mr. M. sitting below stairs: he had dressed with very little assistance, and walked from his chamber by himself. His pulse was, however, not improved, and his breathing very little improved from the morning."

The above history of the form of Mr. M.'s present attack will afford such a view of the close of this interesting case as may supersede the necessity of entering into further details of a progress which, with little variation, terminated fatally on the 27th of November.

In detailing these cases, my chief object is to direct the attention of others to counter-irritation, carried to a greater extent than is customary; and at the same time to describe a mode of applying the actual cautery, which, if generally employed, would set aside many of the objections to its use. Much of the pain arising from the actual cautery, is undoubtedly due to the radiation of the caloric from the hot iron; and, when this is obviated, all the other objections are more imaginary than real. If benefit is to be expected from a counter-irritant, the more powerful the impression, the more likely is it to effect the object intended by its employment; and for this purpose the actual cautery is superior to every other application, independent of the rapidity with which the slough is thrown off, when the operation of an issue is required.

---

